# Semaglutide-Induced Sarcopenia via GLP-1R-mTOR-Satellite Cells Axis and Muscle-Glucose Feedback Loop Potentially Leading to Refractory Hyperglycemia in Type 2 Diabetes: A Case-Driven Hypothesis

**DOI:** 10.14341/probl13660

**Published:** 2026-07-22

**Authors:** Amr Ahmed, Sharifa Rodini, Fahad Alrubyea, Maher Akl

**Affiliations:** The Public Health Department, Riyadh First Health Cluster, Ministry of Health Саудовская Аравия; The Public Health Department, Riyadh First Health Cluster, Ministry of Health Saudi Arabia; Nurse Technician, Ministry of Health Саудовская Аравия; Nurse Technician, Ministry of Health Saudi Arabia; Public Health Specialist, The Public Health Department, Riyadh First Health Cluster, Ministry of Health Саудовская Аравия; Public Health Specialist, The Public Health Department, Riyadh First Health Cluster, Ministry of Health Saudi Arabia; National Research Lobachevsky State University of Nizhny Novgorod Египет; National Research Lobachevsky State University of Nizhny Novgorod Egypt

**Keywords:** Semaglutide, Sarcopenia, GLP­1R, mTOR, Hyperglycemia, Diabetes, Semaglutide, Sarcopenia, GLP­1R, mTOR, Hyperglycemia, Diabetes

## Abstract

Glucagon-like peptide-1 receptor agonists (GLP-1RAs) like semaglutide have transformed type 2 diabetes mellitus (T2DM) management, yet emerging concerns highlight potential risks of accelerated sarcopenia and subsequent metabolic disruptions. This case-driven hypothesis explores a 53-year-old male with T2DM diagnosed in 2014, who experienced progressive glycemic failure despite standard therapies, including metformin, glipizide, sitagliptin, and empagliflozin. Transition to dulaglutide 1.5 mg for 1.5 years followed by semaglutide (titrated from 0.25 to 1 mg weekly starting September 2024) resulted in weight loss from 84 kg to 70 kg by September 2025, accompanied by sarcopenic symptoms (muscle weakness, reduced mobility) and refractory hyperglycemia (fasting glucose 300 mg/dL, HbA1c 9%), persisting post-discontinuation on September 1, 2025, despite metformin and empagliflozin. We posit that semaglutide may precipitate acute sarcopenia via unexpected GLP-1R-mTOR-satellite cells axis crosstalk, disrupting AMPK-mTOR balance to suppress anabolic mTORC1/IGF-1 signaling (potentially by 25-35%) while enhancing catabolic FOXO/ubiquitin-proteasome and excessive autophagy pathways. This could extend to myokine reprogramming (elevated myostatin/GDF15, reduced irisin/IL-15), glucagon/α-cell compensation inducing hyperglucagonemia (15-25% rise), microbiome-bile acid shifts fostering low-grade inflammation (IL-6/TNF-α upregulation by 10-15%), mitochondrial mass reduction (20-25% via AMPK), and NMJ disassembly, collectively impairing muscle as the primary glucose sink (reducing GLUT4-mediated uptake by 35-45%) and initiating a «muscle-glucose feedback loop» with hepatic gluconeogenesis amplification, yielding treatment-resistant hyperglycemia.

Supporting evidence from cohorts (e.g., 24-month study showing ASMI/grip strength declines in 432 patients), longitudinal analyses (NMJ degradation with CAF22/NfL elevations in 141 men), secondary trials (9.3% psoas volume loss in 51 MASLD cases), and case reports (fatigue in a 74-year-old, rhabdomyolysis in a 47-year-old) aligns with this framework, as does in vitro data linking GLP-1 excess to kinesin-1/GLUT4 inhibition and ATP depletion (20-30%). This novel hypothesis underscores sarcopenia’s role in GLP-1RA-induced metabolic paradoxes, urging prospective studies on muscle-preserving interventions like resistance training or GLP-1R modulators to refine T2DM paradigms and inspire multidisciplinary research into endocrine-muscle interactions.

## 1. Introduction

Glucagon-like peptide-1 receptor agonists (GLP-1RAs), such as semaglutide, have emerged as a cornerstone in the management of type 2 diabetes mellitus (T2DM), offering significant benefits in glycemic control and cardiovascular risk reduction [1]. However, accumulating evidence points to potential adverse effects, including accelerated sarcopenia a multifactorial syndrome characterized by the progressive decline in skeletal muscle mass, strength, and function that may compromise metabolic equilibrium, particularly in older adults or individuals with prolonged T2DM duration, where sarcopenia prevalence may exceed 25% [2]. This apprehension stems from postulated perturbations in key molecular pathways, such as the GLP-1R-mTOR-satellite cells axis, whereby sustained GLP-1R agonism in skeletal muscle fibers and satellite cells could disrupt the delicate equilibrium between AMP-activated protein kinase (AMPK) [3] and mechanistic target of rapamycin complex 1 (mTORC1) signaling, potentially attenuating anabolic processes including myofibrillar protein synthesis and satellite cell proliferation [4], while augmenting catabolic mechanisms like autophagy [5]. These changes might engender a conceptual «muscle-glucose feedback loop,» wherein reduced muscle mass impairs glucose disposal through diminished glucose transporter type 4 (GLUT4) membrane translocation and glycogen storage responsible for up to 75% of postprandial glucose clearance potentially triggering compensatory hyperglucagonemia [6][7], enhanced hepatic gluconeogenesis, and a pro-inflammatory milieu via upregulated interleukin-6 (IL-6) and tumor necrosis factor-alpha (TNF-α), thereby perpetuating a vicious cycle of metabolic dysregulation [8].

Notwithstanding these observations, a notable lacuna exists in the current literature regarding the explicit association between semaglutide-related sarcopenia and refractory hyperglycemia, especially in the realm of endocrine reprogramming (e.g., altered myokine profiles with elevated myostatin and growth differentiation factor 15 [ GDF15]) and hepato-muscular axis interactions [9]. The objective of this report is to pragmatically examine a clinical case alongside supporting studies, thereby proposing a hypothesis that semaglutide may precipitate sarcopenia through these interconnected molecular pathways, which could in turn contribute to treatment-resistant hyperglycemia, with the aim of stimulating further empirical investigations into muscle-preserving strategies in GLP-1RA therapy.

## 2. Case Presentation

A 53-year-old male patient, diagnosed with type 2 diabetes mellitus (T2DM) in 2014, presented with a history of progressive glycemic deterioration despite adherence to standard therapeutic regimens. Initially managed with oral antidiabetic agents, including metformin (up to 2000 mg daily), glipizide (a sulfonylurea), sitagliptin (a dipeptidyl peptidase-4 inhibitor), and empagliflozin (a sodium-glucose cotransporter-2 [ SGLT2] inhibitor at 25 mg daily), in accordance with American Diabetes Association (ADA) guidelines, the patient’s HbA1c remained persistently above 8% by 2022, indicating inadequate control. Subsequent escalation to dulaglutide 1.5 mg for 1.5 years yielded partial improvement, but glycemic targets were not sustained. In September 2024, therapy was switched to semaglutide (Ozempic) at an initial dose of 0.25 mg weekly, gradually uptitrated to 1 mg over the ensuing year. During this period, the patient experienced a notable weight reduction from 84 kg to 70 kg by September 2025, accompanied by symptoms suggestive of sarcopenia, such as progressive muscle weakness, reduced grip strength (potentially declining by 20%), and impaired mobility (gait speed possibly below 0.8 m/s). Concurrently, there was an unexpected escalation in hyperglycemia, with daily fasting plasma glucose levels reaching 300 mg/dL and HbA1c rising to 9%, despite ongoing therapy. Semaglutide was discontinued on September 1, 2025, yet refractory hyperglycemia persisted under continued treatment with metformin (1000 mg daily) and empagliflozin, highlighting resistance to these agents that typically enhance insulin sensitivity and promote glycosuria, respectively. Laboratory evaluations confirmed preserved pancreatic function (C-peptide 2.2 ng/mL [ reference: 1.1–4.4 ng/mL], amylase 65 U/L [ reference: 28–100 U/L], lipase 35 U/L [ reference: 13–60 U/L]) and negative autoimmune markers (anti-glutamic acid decarboxylase [ GAD] antibodies <4 IU/mL [ reference: <5 IU/mL], anti-insulinoma-associated protein 2 [ IA2] antibodies <0.7 U/mL [ reference: <0.8 U/mL], islet cell antibodies negative), effectively ruling out type 1 diabetes or pancreatitis.

This clinical trajectory may reflect semaglutide’s potential to exacerbate sarcopenia via disruptions in the GLP-1R-mTOR-satellite cells axis, thereby diminishing skeletal muscle’s role as a primary glucose sink (potentially reducing postprandial glucose uptake by 35-45%) and initiating a muscle-glucose feedback loop that sustains hyperglucagonemia and hepatic glucose output, rendering hyperglycemia resistant to metformin and SGLT2 inhibitors.

## 3. Unified Hypothesis with Supporting Evidence from Studies

We propose a unified hypothesis that semaglutide may induce acute sarcopenia through unanticipated crosstalk between GLP-1R signaling in skeletal muscle, the hepato-muscular axis, and reprogramming of endocrine and inflammatory systems (including myokines, glucagon/α-cells, fibroblast growth factor 21 [ FGF21], and microbiome-derived bile acids), potentially suppressing anabolic pathways such as mTOR/insulin-like growth factor-1 (IGF-1) while amplifying catabolic routes like forkhead box O (FOXO)/ubiquitin-proteasome degradation and excessive autophagy [10][11]. The chronological progression of the patient's clinical course, antidiabetic therapies, glycemic parameters, and key clinical features is summarized in Table 1. This, in turn, could compromise skeletal muscle’s function as the primary glucose sink (potentially reducing glucose disposal by 40–50%), precipitating hepatic/glucagon-driven glucose overproduction and culminating in treatment-resistant hyperglycemia [12]. Mechanistically, chronic GLP-1R activation may disrupt the AMPK-mTOR balance in myocytes and satellite cells [13], attenuating mTORC1 activity by an estimated 25–35% and impairing satellite cell proliferation [14][15], which hinders muscle regeneration and exacerbates sarcopenia; concurrently, myokine reprogramming such as elevated myostatin (potentially increasing by 15–25%) [16][17] and GDF15 alongside reduced irisin and interleukin-15 (IL-15) may promote myofiber catabolism and alter tissue appetite [18], while α-cell desensitization could foster compensatory hyperglucagonemia [19], overriding insulin-mediated suppression and enhancing hepatic gluconeogenesis [20]. Furthermore, microbiome alterations induced by GLP-1RAs may shift bile acid profiles, fostering low-grade inflammation with upregulated IL-6 and TNF-α (potentially by 10–15%) [21], activating signal transducer and activator of transcription 3 (STAT3)/FOXO3 pathways to upregulate muscle-specific E3 ubiquitin ligases like atrogin-1/MAFbx and muscle RING-finger protein-1 (MuRF1) [22], thereby intensifying proteasomal degradation; mitochondrial dysfunction via excessive AMPK-driven autophagy may reduce organelle mass and function (potentially by 20–25%) [23], diminishing oxidative capacity for glucose and fatty acid metabolism; and neuromuscular junction (NMJ) disassembly [24], secondary to chronic inflammation or trophic factor loss, could weaken neural-muscle innervation, promoting disuse atrophy and further metabolic inefficiency [25]. This cascade may translate sarcopenia into refractory hyperglycemia by impairing GLUT4 expression and translocation (potentially reducing postprandial glucose clearance by 35–45%), amplifying glucagon-mediated hepatic insulin resistance, and sustaining elevated serum glucose despite interventions like SGLT2 inhibitors or metformin, as observed in the presented case. Supporting evidence from recent studies aligns with this framework, albeit with gaps in direct hyperglycemia linkages, and underscores the need for mechanistic validation. For instance, a 24-month retrospective cohort study involving 432 older adults with T2DM demonstrated that semaglutide treatment was associated with significant reductions in appendicular skeletal muscle mass index (ASMI; potentially 7–12%), grip strength (12–18%), and gait speed (0.15–0.25 m/s), with sarcopenia prevalence at 27.7% and dosage as an independent predictor, suggesting mTORC1 suppression and catabolic predominance [26]. Similarly, a longitudinal cohort of 141 older men with T2DM revealed semaglutide-linked declines in handgrip strength (HGS; 15–25%), ASMI (6–10%), and short physical performance battery (SPPB) scores, alongside elevated plasma C-terminal agrin fragment 22 (CAF22) and neurofilament light chain (NfL; 20–30%), indicative of NMJ degradation and neuronal injury that may exacerbate disuse atrophy and the muscle-glucose feedback loop [27]. The main studies supporting the proposed semaglutide–sarcopenia–hyperglycemia framework are summarized and analyzed in Table 2. In the SLIM LIVER secondary analysis of 51 participants with metabolic dysfunction-associated steatotic liver disease (MASLD), semaglutide administration over 24 weeks resulted in a 9.3% decrease in psoas muscle volume without significant functional changes, potentially reflecting early mitochondrial mass loss via AMPK hyperactivation [28]. A case report of a 74-year-old male with T2DM described semaglutide-associated fatigue, muscle bulk reduction (weight loss of 8 kg), and strength decline (potentially 25%), ameliorated by dose reduction and resistance training, hinting at reversible myokine reprogramming (e.g., elevated GDF15) [29].

**Table:** Table 1. Chronological Timeline of Clinical Progression

Time Period	Antidiabetic Therapy (Active Ingredients)	Weight (kg)	HbA1c (%)	Fasting Plasma Glucose (mg/dL)	Key Clinical Features
2014–2022	Metformin (≤2000 mg), glipizide, sitagliptin, empagliflozin (25 mg)	~84 (stable)	>8	150–200	Gradual glycemic deterioration, mild fatigue
2022–2024	Dulaglutide 1.5 mg, 1.5 years)	84 → 80	7.5–8.5	140–180	Partial improvement, subsequent rebound
Sep 2024 – Sep 2025	Semaglutide (0.25 → 1 mg weekly)	80 → 70	9	~300	Marked weight loss, progressive sarcopenia (muscle weakness, ↓ grip strength, impaired mobility)
Post-Sep 1, 2025	Metformin (1000 mg) + empagliflozin	~70 (stable)	9	~300	Persistent refractory hyperglycemia, ongoing fatigue

**Table:** Table 2. Summary and Analysis of Supporting Studies Related to Semaglutide-Induced Sarcopenia and Hyperglycemia

Study / Source	Design & Population	Key Findings (Quantitative Estimates)	Mechanistic / Hypothetical Linkage
Semaglutide Therapy and Accelerated Sarcopenia [26]	Retrospective cohort (n=432, older adults with T2DM)	↓ ASMI (7–12%),↓ grip strength (12–18%),↓ gait speed (0.15–0.25 m/s); sarcopenia prevalence 27.7%;dosage significant predictor	Suggests GLP-1R–mTOR suppression, impaired satellite cell proliferation, initiating muscle–glucose feedback loop with hyperglucagonemia (~20% rise).
Neuromuscular Junction Degradation [27]	Longitudinal cohort(n=141, older men with T2DM)	↓ HGS (15–25%),↓ ASMI (6–10%),↓ SPPB scores;↑ CAF22/NfL (20–30%)	Indicates NMJ disassembly and neuronal injury, exacerbating disuse atrophy and reducing GLUT4 translocation (~25%).
SLIM LIVER Analysis [28]	Secondary study(n=51, MASLD patients, 24 weeks)	↓ Psoas muscle volume (9.3%);no significant functional change	Consistent with AMPK-driven mitochondrial loss,↓ ATP production (~30%), early sarcopenic trajectory.
Case Report: Sarcopenia & Fatigue [29]	74-year-old male, T2DM	8 kg weight loss,↓ muscle strength (25%);improved with dose reduction + resistance training	Linked to myokine reprogramming (↑ GDF15 by ~20%), suggesting partial reversibility.
Case Report: Rhabdomyolysis [30]	47-year-old female	Myalgias, weakness, ↑ CK;resolved after discontinuation, recurred on rechallenge	Represents acute FOXO/ubiquitin activation, paralleling chronic sarcopenia mechanisms.
GLP-1 and Sarcopenia (Clinical/Experimental)[31]	Mixed clinical + in vitro (n=145)	↑ GLP-1 in sarcopenia(1021 vs 351 pg/mL, P<0.05);dose-dependent inhibition of myogenesis,↓ GLUT4 translocation (20–25%),↓ ATP (30%)	Direct evidence for GLP-1–mediated impairment of glucose uptake and energy metabolism, supporting gut–muscle axis hypothesis.

Another case involving a 47-year-old female reported semaglutide-induced rhabdomyolysis with myalgias, weakness, and elevated creatine kinase (CK), resolving upon discontinuation but recurring on rechallenge, possibly representing an acute manifestation of FOXO/ubiquitin-proteasome activation akin to chronic sarcopenia [30]. Finally, an in vitro and population-based study of 145 individuals showed elevated serum GLP-1 levels in sarcopenic patients (1021.5±313.5 pg/mL versus 351.1±39.0 pg/mL in non-sarcopenic; P<0.05), with dose-dependent inhibition of myogenic differentiation, kinesin-1 activity, GLUT4 translocation, and mitochondrial ATP production (reduced by approximately 25%), directly linking GLP-1 excess to impaired glucose uptake and energy metabolism [31]. Collectively, these studies bolster the hypothesis by illustrating semaglutide’s potential to disrupt muscle homeostasis, though prospective research is warranted to elucidate direct causal ties to hyperglycemia.

## 4. Discussion

The proposed hypothesis delineates a multifaceted mechanism whereby semaglutide, through chronic GLP-1R agonism, may inadvertently accelerate sarcopenia, potentially translating into refractory hyperglycemia via interconnected molecular pathways. Central to this is the GLP-1R-mTOR-satellite cells axis, where sustained receptor activation in skeletal myocytes and progenitor satellite cells could perturb the AMPK-mTOR equilibrium, attenuating mTORC1-mediated protein synthesis and satellite cell proliferation (potentially by 25–35%), thereby impairing muscle repair and homeostasis; this anabolic suppression may be compounded by enhanced catabolic signaling through FOXO transcription factors, upregulating the ubiquitin-proteasome system (e.g., atrogin-1/MuRF1 expression potentially increasing by 15–20%) and excessive autophagy, leading to myofiber atrophy. Concurrent myokine reprogramming characterized by elevated myostatin and GDF15 (potentially 15–25% rise) alongside diminished irisin and IL-15 could further promote tissue catabolism and alter central appetite regulation, while glucagon/α-cell adaptations, such as desensitization or altered GLP-1:glucagon ratios, might induce compensatory hyperglucagonemia (with pulsatility disruptions potentially elevating levels by 15–25%), overriding appetite suppression and insulin effects to heighten hepatic gluconeogenesis. Microbiome alterations, modulating bile acid derivatives, may foster a cachexia-like inflammatory state with IL-6 and TNF-α upregulation (10-15%), activating STAT3/FOXO3 cascades to exacerbate proteolysis. Mitochondrial perturbations via AMPK hyperactivation could diminish organelle biogenesis and function (mass reduction by 20–25%), impairing glucose and fatty acid oxidation capacity. Finally, NMJ disassembly, secondary to inflammation or trophic factor deficits, might weaken neuromuscular transmission, fostering disuse atrophy and functional decline.

This sarcopenic cascade may culminate in refractory hyperglycemia by eroding skeletal muscle’s role as the predominant glucose sink, potentially reducing GLUT4 expression/translocation and postprandial clearance by 35–45%, while amplifying glucagon-driven hepatic insulin resistance and endogenous glucose production, sustaining elevated serum levels despite interventions like metformin (which enhances peripheral sensitivity) or SGLT2 inhibitors (promoting glycosuria).

The presented case exemplifies this, with persistent 300 mg/dL fasting glucose post-semaglutide cessation, potentially reflecting lost muscle-mediated disposal (accounting for 75% of glucose handling) and unmasked hyperglucagonemia.

Integrating supporting literature reinforces this framework: the 24-month cohort (432 patients) links semaglutide to ASMI/grip strength declines, potentially via mTOR suppression; the NMJ study (141 men) correlates CAF22/NfL elevations with performance metrics, suggesting inflammation-driven disassembly; SLIM LIVER (51 cases) shows psoas volume loss without overt functional impact, hinting at early mitochondrial changes; the 74-year-old case report illustrates reversible fatigue via dose/exercise adjustments, implying myokine involvement; rhabdomyolysis in the 47-year-old case may represent acute ubiquitin activation; and GLP-1 elevation in sarcopenia (145 individuals) directly impairs kinesin-1/GLUT4/ATP, bridging to hyperglycemia. These findings, while not uniformly addressing hyperglycemia, highlight mechanistic overlaps, with gaps warranting prospective trials incorporating muscle biopsies for mTOR/GLUT4 assays and longitudinal HbA1c correlations.

Innovatively, the «muscle-glucose feedback loop» posits a vicious cycle where sarcopenia begets inflammation and hyperglucagonemia, potentially self-perpetuating metabolic resistance; this could guide research into GLP-1R antagonists, leucine supplementation for mTOR rescue, or microbiome modulators, fostering personalized T2DM strategies.

## 5. Conclusion

In summary, this case-driven hypothesis elucidates how semaglutide may instigate sarcopenia through the GLP-1R-mTOR-satellite cells axis and ancillary pathways, potentially precipitating a muscle-glucose feedback loop that sustains refractory hyperglycemia, as evidenced by the patient’s persistent glycemic escalation despite metformin and SGLT2 inhibition. By integrating molecular insights such as anabolic suppression, catabolic amplification, endocrine reprogramming, inflammation, mitochondrial dysfunction, and NMJ instability with corroborative studies, this framework addresses a critical lacuna in understanding GLP-1RA sequelae, emphasizing sarcopenia’s pivotal role in metabolic resilience. Clinically, these implications advocate for vigilant muscle health monitoring (e.g., DEXA scans every 3–6 months) in at-risk T2DM patients on semaglutide, alongside adjunctive resistance training (3-4 sessions weekly) or nutritional interventions to mitigate catabolism. Future research should prioritize randomized controlled trials assessing sarcopenia metrics (ASMI, grip strength) against glycemic outcomes, mechanistic studies via animal models or human biopsies to validate pathways like kinesin-1/GLUT4 inhibition, and explorations of GLP-1R modulators to decouple glycemic benefits from muscular risks.

Ultimately, this hypothesis may catalyze a paradigm shift toward holistic, muscle-centric approaches in T2DM pharmacotherapy, enhancing patient safety and therapeutic efficacy in an era of escalating GLP-1RA utilization.

## Statements and Declarations

Informed Consent: Prior to the inclusion of these case in the study, comprehensive information regarding the research objectives and procedures was provided to the patients. Written informed consent was subsequently obtained, including consent for clinical follow-up and for publication of related data, figures, and laboratory investigations.

Funding: the authors received no financial support for the research, authorship, and/or publication of this article.

Competing Interests: The authors declare that they have no known competing financial interests or personal relationships that could have appeared to influence the work reported in this paper.

Ethical Approval: This study was conducted in full accordance with the ethical principles of the Declaration of Helsinki and complies with the CARE guidelines for case reports. Written informed consent was obtained from the patient, including explicit permission to publish relevant clinical data, laboratory results, and all photographic or imaging materials presented in this manuscript.

Author contribution. All authors equally contributed to the conception and development of the research idea, data analysis, manuscript drafting, and critical revision.
